# Synergistic Suppression of Secondary Electron Yield from Al_2_O_3_ Ceramic Windows by TiN Film and Laser Surface Texturing

**DOI:** 10.3390/nano16090513

**Published:** 2026-04-24

**Authors:** Baolong Ma, Shixi Chen, Chen Chen, Fanxi Zhang, Yaru Wang, Yixin Si, Jinglun Li, Jinghe Yang, Haipeng Li, Sheng Wang, Yupeng Xie

**Affiliations:** 1 Shaanxi Engineering Research Center of Advanced Nuclear Energy, School of Nuclear Science and Technology, School of Energy and Power Engineering, Xi’an Jiaotong University, Xi’an 710049, China; baolongma@xjtu.edu.cn (B.M.);; 2 Shaanxi Key Laboratory of Advanced Nuclear Energy and Technology, School of Nuclear Science and Technology, School of Energy and Power Engineering, Xi’an Jiaotong University, Xi’an 710049, China; 3 State Industry-Education Integration Center for Medical Innovations, Xi’an Jiaotong University, Xi’an 710049, China; 4 Huzhou Neutron Science Laboratory of Xi’an Jiaotong University, Science Valley Medium-Sized Building #1, Huzhou 313000, China; 5 China Institute of Atomic Energy, Beijing 102413, China; 6 HBNCT Co., Ltd., Hangzhou 310000, China

**Keywords:** Al_2_O_3_ ceramic windows, TiN film, laser surface texturing, SEY, synergistic suppression

## Abstract

To suppress the Secondary Electron Yield (SEY) of Al_2_O_3_ ceramic surfaces for accelerator ceramic windows, a synergistic strategy integrating TiN film deposition and laser surface texturing was developed. TiN films were first deposited on Al_2_O_3_ substrates by pulsed DC magnetron sputtering, and the sputtering power was optimized through systematic characterization of the film morphology and chemical states, with 300 W identified as the optimal deposition condition. Laser surface texturing was then introduced to construct micro-structured Al_2_O_3_ surfaces with different geometrical features. Among the investigated laser powers, the 12 W-treated surface exhibited the most developed surface morphology and the highest roughness, indicating the most favorable topography for electron trapping. SEY measurements showed that the maximum SEY decreased from 8.2 for the as-received Al_2_O_3_ to 5.5 after deposition of a 10 nm TiN film, and was further reduced to 2.1, 1.0, and 1.7 for the textured TiN/Al_2_O_3_ surfaces prepared at 6, 12, and 18 W, respectively, with the best suppression for the 12 W textured TiN/Al_2_O_3_. The enhanced performance is attributed to the synergistic effect of low-SEY TiN surface chemistry and geometrical electron trapping induced by laser texturing. This work provides an effective route for constructing low-SEY Al_2_O_3_ ceramic surfaces for beam-window-related applications.

## 1. Introduction

In high-power accelerators and high-vacuum radio-frequency systems, the interaction between charged particles, electromagnetic fields, and material surfaces can lead to the accumulation and multiplication of low-energy electrons, thereby inducing electron cloud formation or multipactor discharges. These phenomena may further cause beam instability, RF power degradation, local overheating, vacuum deterioration, and even surface breakdown. Therefore, the Secondary Electron Yield (SEY) is widely recognized as a key surface parameter governing the operational stability of such devices [1,2]. The multipacting effect is a resonant phenomenon occurring in RF vacuum devices, in which electrons collide with surfaces under a periodic electric field. When the SEY exceeds 1 within a specific incident energy range, an exponential increase in electron population can occur, potentially leading to discharges and component damage [3]. In high-power continuous-wave operation, power couplers are particularly susceptible to multipacting between the inner and outer conductors on the vacuum side. Therefore, for accelerator vacuum components, reducing the SEY of critical inner surfaces has been demonstrated to be an effective strategy for suppressing electron multiplication and improving long-term reliability [1].

Al_2_O_3_ ceramics are widely used in high-power vacuum windows, RF coupling windows, and related insulating structures because of their excellent electrical insulation, high thermal stability, good mechanical strength, and relatively low dielectric loss [4]. However, Al_2_O_3_ surfaces generally exhibit relatively high SEY, making them susceptible to electron-induced phenomena such as multipactor, surface charging, and local breakdown under electron bombardment conditions. Early studies on alumina RF windows revealed that window failure is closely associated with secondary-electron-induced surface heating and electron multiplication [5,6]. In addition, surface charging and potential buildup can further aggravate the service instability of ceramic windows. Subsequent investigations confirmed that the SEY and charging behavior of alumina are among the dominant factors controlling the power-handling capability and failure threshold of such components [7].

To address the high-SEY characteristics of ceramic surfaces, TiN films have long been considered one of the most representative anti-multipactor materials. Since the 1990s, TiN films have been applied to alumina RF windows to suppress electron emission and mitigate multipactor [8]. Later studies further demonstrated that TiN films can significantly reduce the SEY of alumina ceramics, and that the suppression effect strongly depends on the film thickness [9]. Ogura et al. reported that nanometer-scale TiN films can effectively suppress the SEY of alumina-based surfaces [10]. Once the films reach sufficient surface coverage, the SEY can be substantially decreased, in some cases approaching unity. Moreover, TiN and other anti-multipactor materials have been reported to retain favorable electron-suppression capability even at elevated temperatures, indicating their potential for stable engineering applications [11]. Therefore, optimizing the deposition parameters and thickness of TiN films on Al_2_O_3_ ceramic surfaces represents a practical and effective route for constructing low-SEY functional surfaces.

In addition to modifying the intrinsic electron-emission characteristics of a surface through functional coatings, surface geometry engineering has also been proven to be an effective approach for reducing the effective SEY. The underlying mechanism is that, when the surface is patterned with grooves, pores, pits, or other textured microstructures, the emitted secondary electrons are more likely to undergo multiple collisions, reflections, and re-absorption before escaping into the vacuum. As a result, the number of electrons that finally leave the surface is significantly reduced. Pivi et al. experimentally and theoretically demonstrated a pronounced reduction in SEY on grooved surfaces [12]. Ye et al. further achieved effective SEY suppression using micro-porous array structures [13], while Sattler et al. clarified the geometrical mechanism by which porous morphologies suppress SEY and multipactor [14]. These studies indicate that surface micro-structuring provides a regulation pathway distinct from conventional low-SEY coatings, relying primarily on electron trapping enabled by geometrical effects.

In recent years, laser surface engineering has emerged as a promising method for SEY reduction in accelerator-related applications because of its non-contact nature, high controllability, compatibility with a wide range of materials, and ability to fabricate multiscale surface textures. Valizadeh et al. demonstrated that laser ablation surface engineering can effectively reduce SEY for electron-cloud mitigation [15]. Calatroni et al. systematically investigated the optimization of SEY on laser-structured copper surfaces [16]. Moreover, Nivas et al. showed that laser-induced periodic structures and the surface morphologies formed under different processing environments can significantly influence SEY [17]. Overall, laser texturing can endow a surface with strong electron-trapping capability by creating high-aspect-ratio microstructures or multiscale rough interfaces, and is therefore regarded as an important strategy for fabricating low-SEY surfaces.

Although both TiN coatings and laser surface texturing have shown considerable potential for suppressing SEY, most existing studies have focused on metallic substrates, single coating modifications, or single structural regulation strategies. Systematic studies on the combined regulation of SEY on Al_2_O_3_ ceramic beam-window surfaces through low-SEY films and laser-induced microstructures remain limited. In this work, Al_2_O_3_ ceramic beam-window substrates were selected as the model system. TiN film deposition parameters were first optimized by pulsed DC magnetron sputtering. Laser surface texturing was then introduced onto the Al_2_O_3_ substrates, followed by the deposition of TiN films with different thicknesses under the optimized sputtering conditions. By combining surface morphology characterization, structural analysis, and SEY measurements, the synergistic regulation mechanism of TiN films and laser-induced surface textures on the SEY of Al_2_O_3_ ceramic windows was systematically investigated. This study is expected to provide both experimental evidence and theoretical support for the design of low-SEY functional surfaces for accelerator beam-window applications.

## 2. Materials and Methods

### 2.1. Materials

The 99% Al_2_O_3_ ceramics used in this study were purchased from Qianrui Ceramic Distributor, Suzhou Industrial Park, China, and cut into specimens with dimensions of 10 × 10 × 1 mm. Prior to film deposition, all samples were sequentially cleaned by ultrasonication in acetone, absolute ethanol, and ultrapure water to remove surface contaminants and residual oil. After cleaning, the samples were dried in an oven at 80 °C overnight and subsequently used as substrates. Acetone and absolute ethanol (purity ≥ 99%) were purchased from Sinopharm Chemical Reagent Co., Ltd. (Shanghai, China). The TiN sputtering target was supplied by Element Tech Material Co., Ltd. (Weihai, China). The target had dimensions of 420 × 80 × 4 mm, a purity of 99.9%, and was bonded to a 428 × 88 × 4 mm copper backing plate.

### 2.2. Laser Surface Texturing

Laser surface texturing was carried out on the Al_2_O_3_ ceramic substrates using a K20-CS nanosecond pulsed fiber laser system (Han’s Laser, Shenzhen, China) to generate surface microstructures. The system was equipped with a 20 W IPG laser source, a galvanometer scanner, an EMCC control unit, a mechanical stage, a workstation, and the corresponding control software. The main processing parameters of the laser system included a maximum output power of 20 W, a pulse width of 100 ns, a repetition rate of 20 kHz, a spot diameter of 15 μm, and a wavelength of 1064 nm. To investigate the influence of laser power on the ablation behavior of the Al_2_O_3_ surface, as well as its subsequent effect on TiN film deposition and SEY, laser texturing was performed at three power levels: 6 W, 12 W, and 18 W, selected as the main variable, while all other processing parameters were kept constant to ensure a controlled comparison. After laser treatment, the samples were ultrasonically cleaned in acetone and ultrapure water for 30 min to remove residual debris generated during ablation. The cleaned samples were then dried in an oven at 80 °C for 12 h before further use.

### 2.3. TiN Film by Pulsed DC Magnetron Sputtering

TiN films were deposited using a JCPF1600 pulsed DC magnetron sputtering system manufactured by Beijing Technol Science Co., Ltd. (Beijing, China). To minimize the influence of residual gases on the sputtering process, the chamber atmosphere, particularly residual H_2_O and O_2_, was carefully controlled. Before each sputtering experiment, Al_2_O_3_ ceramic substrates were mounted onto the sample holder using a high-temperature conductive adhesive. The holder was then transferred into the sputtering chamber using a sample transfer cart. Prior to deposition, the chamber was evacuated to a base pressure of 8 × 10^−4^ Pa. To optimize the sputtering conditions for TiN films, the sputtering pressure was fixed at 0.5 Pa, the Ar flow rate was maintained at 20 sccm, and the duty cycle was set to 90%. Sputtering power, which was considered one of the most influential parameters, was systematically varied at 100, 200, 300, 400, and 500 W. During deposition, the sample holder was positioned directly in front of the TiN target. Once the desired film thickness was achieved, the chamber pressure was gradually restored to match that of the glove box, which was maintained slightly above atmospheric pressure. After the optimized deposition parameters were determined, TiN films with a thickness of 10 nm were further prepared for SEY measurements. The coated samples were then removed from the chamber for subsequent use. In this work, a TiN film thickness of 10 nm was selected to balance surface chemical modification and preservation of the laser-induced surface morphology.

### 2.4. Secondary Electron Yield

Before testing, the samples were ultrasonically cleaned in absolute ethanol for 10 min and dried in a vacuum oven at 60 °C for 30 min. The SEY was measured by the electron bombardment method on a Gemini SEM 500 (ZEISS, Oberkochen, Germany). All measurements were carried out in a high-vacuum chamber with a pressure below 1 × 10^−5^ Pa to minimize the influence of residual gases. The measurement system consisted of an electron gun, a sample stage, a hemispherical secondary-electron collector, and a data acquisition unit. The primary electron beam energy was varied from 0.1 to 3 keV, with step sizes of 0.05 keV from 0.1 to 1 keV, 0.2 keV from 1 to 2 keV, and 0.5 keV from 2 to 3 keV. During measurement, the primary beam was normally incident on the sample surface, and the primary electron current, together with the secondary electron current, was recorded simultaneously. The SEY was calculated as the ratio of the secondary electron current to the primary electron current. All samples for SEY measurements are listed in Table 1.

### 2.5. Sample Characterizations

Before deposition, a mask line was introduced on a clean Si substrate to create a step edge. After deposition, the coated area was partially removed with acetone to expose the substrate, and the step height was measured to obtain the film thickness, using a surface profilometer (Alphastep D-500, KLA Corporation, Milpitas, CA, USA) operated with a scan length of 4 mm, a scan speed of 0.20 mm s^−1^, and a stylus force of 15.0 mg. Surface morphology was examined using a JEOL JSM-7800F (Tokyo, Japan) scanning electron microscope (SEM) at an accelerating voltage of 5–15 kV. Elemental distribution was analyzed by energy-dispersive spectroscopy (EDS) attached to the SEM to evaluate compositional uniformity. The crystal structure of the films was characterized by X-ray diffraction (XRD) using a Bruker D8 Advance diffractometer with Cu Kα radiation (λ = 1.5406 Å). Diffraction patterns were collected over the 2θ range of 20–90°, with a counting time of 0.15 s per step. The surface chemical composition and bonding states were further investigated by X-ray photoelectron spectroscopy (XPS) using an AXIS UltraDLD system with monochromatic Al Kα radiation (1486.6 eV). The X-ray source was operated at 150 W, and the spot size was 300 × 700 μm^2^. All binding energies were calibrated using the C 1s peak at 284.8 eV. A laser scanning confocal microscope (LSCM, Leica DCM8) was employed to characterize the three-dimensional surface topography of the laser-textured Al_2_O_3_ ceramic substrates and to quantify the corresponding surface roughness.

## 3. Results and Discussion

### 3.1. TiN Film Deposition Optimization for Sputtering Powers

#### 3.1.1. SEM

**Figure 1:**
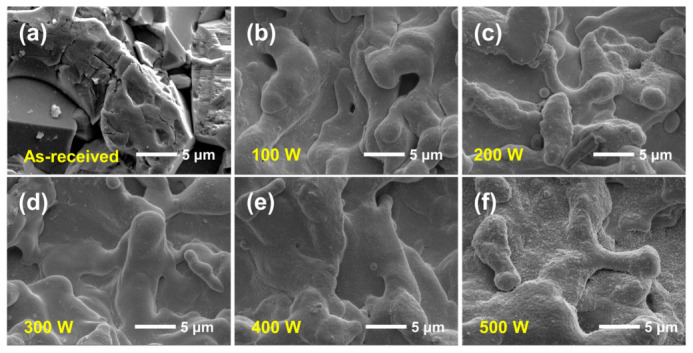
SEM images of the as-received Al_2_O_3_ substrate and TiN films deposited at different sputtering powers: (**a**) as-received Al_2_O_3_, and TiN films deposited at the sputtering powers of (**b**) 100 W, (**c**) 200 W, (**d**) 300 W, (**e**) 400 W, and (**f**) 500 W.

Figure 1 presents the SEM images of the as-received Al_2_O_3_ substrate and the TiN films deposited at sputtering powers from 100 to 500 W. As shown in Figure 1a, the as-received Al_2_O_3_ substrate exhibits a distinctly angular surface with sharp edges and irregular facets. After TiN deposition, the surface morphology became noticeably smoother and more rounded, indicating that the deposited TiN film effectively covered the original ceramic surface and substantially altered its topographical features. This transition from an angular substrate morphology to a more rounded film surface suggests that the deposited species progressively filled and bridged the original surface asperities during film growth.

Although the TiN-coated samples deposited at different sputtering powers show broadly similar overall morphologies, closer examination reveals several important differences. Firstly, the original substrate morphology is no longer directly visible after deposition, confirming that the TiN film formed a continuous covering layer over the Al_2_O_3_ surface. Secondly, locally raised features can be observed at some of the original surface relief positions, implying that film growth preferentially developed on pre-existing topographical sites. In addition, some localized nodular or nearly spherical particles are present on the film surface. Such features are consistent with a growth process involving surface nucleation, island formation, and subsequent coalescence, which is commonly observed in sputtered thin films, especially on topographically non-uniform substrates [18].

At sputtering powers below 300 W (Figure 1b–d), the TiN films appear relatively smooth, and only a limited number of fine surface dots can be identified. It should be noted that the TiN film at this stage mainly acts as a conformal layer that follows and smooths the initial morphology of the Al_2_O_3_ substrate, rather than completely reconstructing the surface topography. By contrast, as the sputtering power increases, the surface gradually becomes rougher, and a larger number of tiny nodular features emerges. This trend is particularly pronounced at 500 W (Figure 1f), where the film surface is covered by a much higher density of fine protrusions. This evolution in morphology is closely related to the variation in deposition rate. As the sputtering power increases, the deposition rate rises significantly (from 0.55 to 5.9 nm/min), leading to a higher arrival flux of sputtered species. Under such conditions, the time available for surface diffusion and relaxation becomes insufficient, which promotes local agglomeration and the formation of nodular features (Table 2). The dependence of film morphology on sputtering power can be attributed to changes in deposition flux, energy transfer, and surface diffusion kinetics. Increasing sputtering power generally enhances the arrival rate of sputtered species and modifies the growth mode of the film, which can promote local agglomeration and roughening when the growth rate becomes too high for sufficient surface relaxation [19]. Similar power-dependent increases in grain size, surface roughness, and morphological heterogeneity have been reported for sputtered films in previous studies [20]. From the viewpoint of surface quality, the SEM results suggest that excessively high sputtering power is unfavorable for obtaining a uniform TiN film. In particular, the film deposited at 500 W exhibits the most pronounced fine-dot or nodular morphology, indicating enhanced surface heterogeneity. In contrast, the films prepared at lower and intermediate powers, especially below 300 W, show fewer such features and comparatively smoother surfaces. Therefore, although all sputtering powers investigated in this work were capable of forming TiN-covered surfaces, moderate sputtering power appears to be more favorable for producing TiN films with improved morphological uniformity, which is expected to be beneficial for subsequent SEY regulation and performance evaluation.

#### 3.1.2. XRD

**Figure 2:**
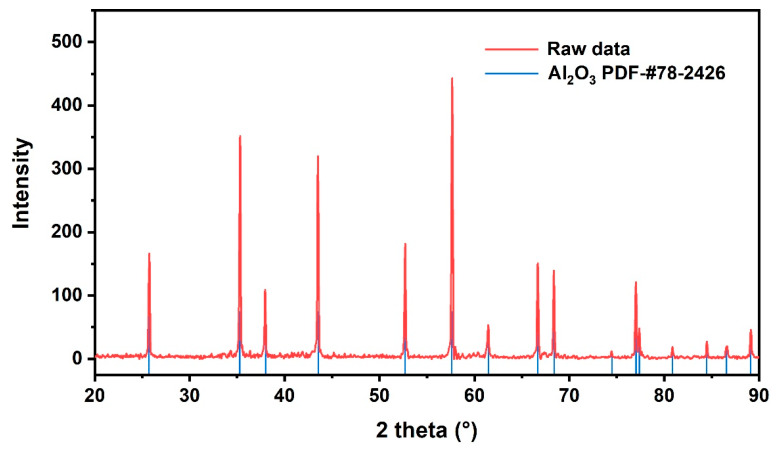
XRD pattern of the TiN-film-coated Al_2_O_3_ substrate deposited at 300 W. All diffraction peaks can be indexed to Al_2_O_3_ (PDF #78-2426), while no detectable TiN diffraction peak is observed.

To evaluate the phase structure after deposition, XRD characterization was carried out using the sample prepared at 300 W as a representative case. As shown in Figure 2, all diffraction peaks can be indexed to the Al_2_O_3_ substrate and are in good agreement with the standard card (PDF #78-2426). No additional diffraction peaks associated with TiN were detected, and the diffraction profile remains essentially identical to that of the as-received Al_2_O_3_ substrate, indicating that the diffraction pattern is dominated by the Al_2_O_3_ substrate under the present measurement conditions. The absence of TiN-related peaks is reasonably attributed to the extremely small film thickness, for which the diffracted intensity of the film is too weak compared with the much stronger reflections from the ceramic substrate. In conventional θ-2θ XRD measurements, diffraction from ultrathin films is often masked by the substrate signal, especially when the deposited layer is only a few nanometers thick or has a limited crystalline volume [21].

#### 3.1.3. XPS

**Figure 3:**
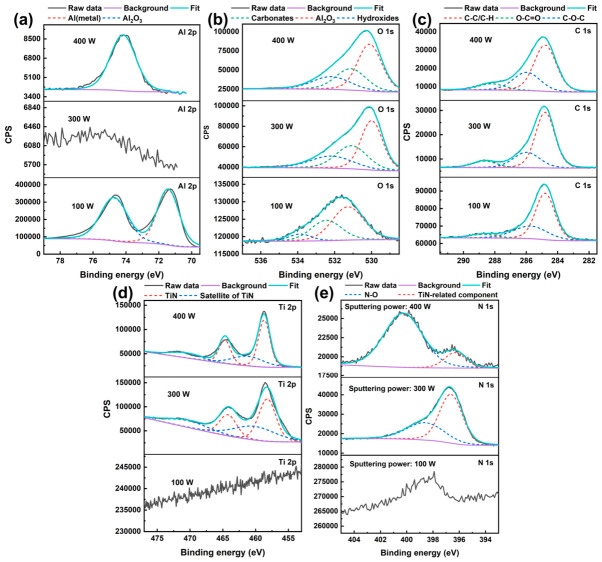
High-resolution XPS spectra of TiN films deposited on Al_2_O_3_ substrates at different sputtering powers: (**a**) Al 2p, (**b**) O 1s, (**c**) C 1s, (**d**) Ti 2p, and (**e**) N 1s. The spectra shown correspond to the representative samples prepared at 100, 300, and 400 W.

Compared with XRD, XPS is much more surface-sensitive and is therefore more suitable for probing ultrathin films whose thickness is within the near-surface analysis depth [22]. In view of this, to further clarify the chemical states of the deposited films and to compensate for the limited sensitivity of conventional XRD to ultrathin layers, XPS measurements were carried out on TiN films prepared at 100, 300, and 400 W, which represent the three typical states observed in this work (Figure 3).

In the Al 2p spectra of Figure 3a, the sample deposited at 100 W can be deconvoluted into two components located at approximately 71.43 eV and 74.7 eV, corresponding to Al(metal) species and lattice Al in Al_2_O_3_, respectively [23]. The simultaneous presence of these two components indicates that the TiN film deposited at 100 W was too thin or discontinuous to fully screen the substrate signal within the XPS probing depth. By contrast, no obvious Al-related peak was detected for the sample deposited at 300 W, suggesting that the substrate surface was effectively covered by the TiN film. For the sample deposited at 400 W, an Al 2p signal reappeared, but it was dominated by the Al_2_O_3_-related component, with relatively high intensity. This result suggests that, although film deposition was achieved at 400 W, the surface may contain defects, pinholes, or locally non-uniform regions, allowing the substrate-related signal to be partially detected again. Such behavior is consistent with the SEM observation that higher sputtering power tended to produce a rougher and more heterogeneous surface morphology.

The O 1s spectra of all three samples can be fitted using three components assigned to carbonates, Al_2_O_3_ lattice oxygen, and hydroxides, centered at approximately 531.1 eV for carbonate, 523.0 eV for Al_2_O_3_, and 532.2 eV for hydroxide, respectively (Figure 3b) [24]. The persistence of these oxygen-related components at all sputtering powers is expected because the Al_2_O_3_ ceramic substrate remains the dominant oxygen-containing phase in the near-surface region. In addition, hydroxyl groups and carbonate species are commonly observed on oxide surfaces after exposure to an ambient atmosphere. Therefore, the O 1s results indicate that the detected surface chemistry still contains substantial contributions from substrate-related oxide species together with adsorbed oxygen-containing contaminants, even after TiN deposition. The C 1s spectra of all samples can be resolved into three components corresponding to C-C/C-H, O-C=O, and C-O-C, located at approximately 284.8 eV for C-C/C-H, 288.7 eV for O-C=O, and 285.9 eV for C-O-C, respectively (Figure 3c) [25]. Among them, the C-C/C-H peak mainly originates from adventitious hydrocarbon contamination and was also used as the reference for binding-energy calibration. The oxygen-containing carbon species are attributed to adsorbed surface contaminants formed during air exposure and sample transfer. Since similar C 1s features are observed for all samples, the carbon signal is considered to arise primarily from surface contamination rather than from the intrinsic TiN film itself.

More direct evidence for successful TiN deposition is provided by the Ti 2p spectra in Figure 3d. For the sample deposited at 100 W, no clear Ti 2p signal was detected, further confirming that the deposited layer under this condition was either too thin or too discontinuous to generate a resolvable Ti-related photoelectron signal. In contrast, the samples deposited at 300 and 400 W both exhibit characteristic Ti 2p doublets associated with TiN, with Ti 2p3/2 and Ti 2p1/2 peaks located at approximately 464.2 eV and 458.2 eV, respectively [26]. In addition, satellite features were observed at approximately 470.3 eV and 460.2 eV, which are characteristic of TiN and are commonly considered in self-consistent fitting of Ti 2p spectra for TiN surfaces [25]. The appearance of the TiN main doublet together with its satellite structure confirms the formation of Ti-N bonding environments in the films deposited at 300 and 400 W. The N 1s spectra in Figure 3e further support this conclusion. No obvious N-related signal was detected for the sample prepared at 100 W, which is fully consistent with the absence of a clear Ti 2p peak and indicates that a chemically identifiable TiN layer was not effectively established under this condition. In contrast, the spectra of the 300 and 400 W samples can be deconvoluted into two components centered at approximately 396.6 eV for TiN-related N and 398.6 eV for N-O. According to the fitting model used in this work, the lower-binding-energy component was assigned to the TiN-related nitrogen species, denoted here as N^3+^, while the higher-binding-energy component corresponds to N-O species. Importantly, the relative contribution of the TiN-related N component is much higher for the 300 W sample, whereas the 400 W sample exhibits a comparatively lower fraction of this component and a more noticeable contribution from N-O species. This indicates that the film deposited at 300 W contains a more dominant Ti-N bonding environment, while the film deposited at 400 W is more affected by surface oxidation, defect-related bonding, or local chemical heterogeneity.

Taken together, the sample deposited at 300 W shows the most favorable combination of features, including effective suppression of the Al signal, clear TiN-related Ti 2p peaks with satellite structure, and a dominant TiN-related N 1s component. From a theoretical perspective, this result can be understood as a balance between film continuity and growth-induced defect formation. At insufficient sputtering power, the flux of sputtered species is too low to rapidly establish a continuous TiN layer on the ceramic surface, leading to incomplete coverage and persistent substrate exposure. At excessively high power, however, the increased deposition rate and energetic particle bombardment can promote non-uniform growth, local roughening, and defect generation, which in turn deteriorate the chemical uniformity of the near-surface region. The intermediate power of 300 W appears to provide the most suitable balance between deposition efficiency and surface stability, enabling the formation of a more continuous and chemically well-defined TiN film.

### 3.2. Microstructure of Laser-Textured Al_2_O_3_ Ceramic After Laser Surface Texturing

#### 3.2.1. SEM

**Figure 4:**
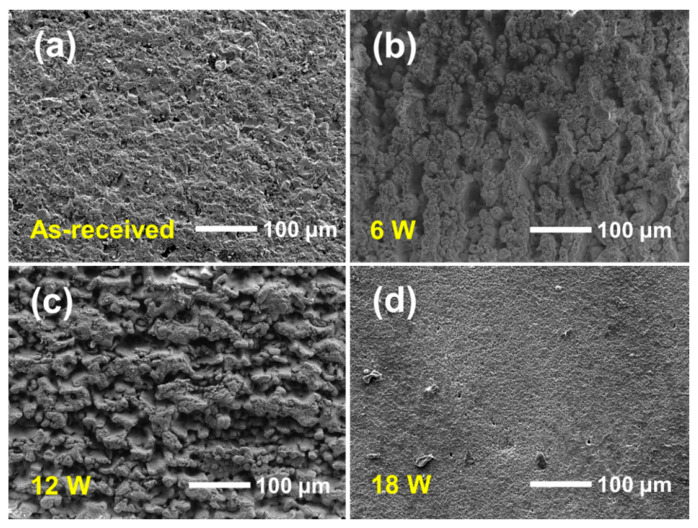
SEM images of the Al_2_O_3_ substrates after laser surface texturing at different laser powers with 10 nm TiN: (**a**) as-received, (**b**) 6 W, (**c**) 12 W, and (**d**) 18 W.

**Figure 5:**
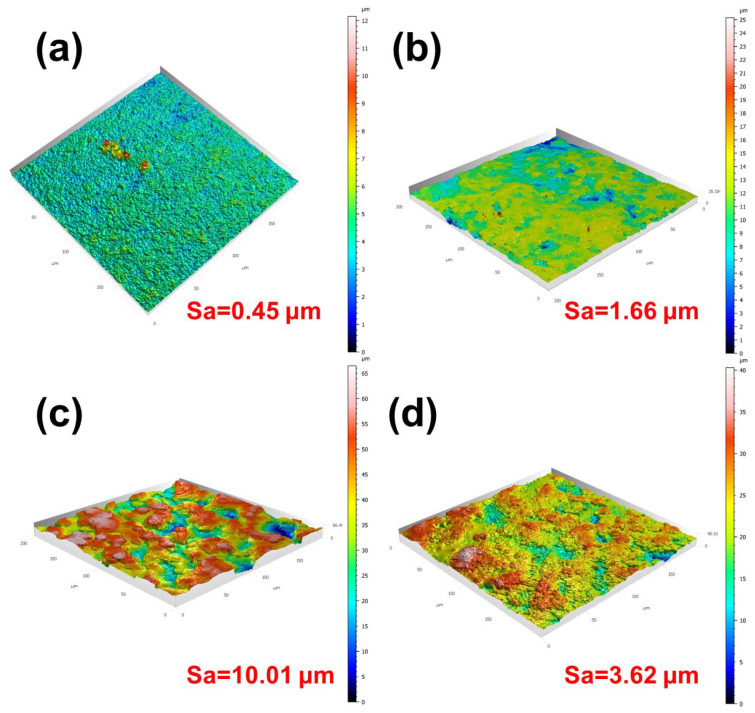
Three-dimensional LSCM images and surface roughness of the Al_2_O_3_ substrates after laser texturing at different laser powers: (**a**) as-received, (**b**) 6 W, (**c**) 12 W, and (**d**) 18 W.

To further reduce the SEY of the Al_2_O_3_ ceramic substrate, laser surface texturing was introduced to construct surface microstructures capable of enhancing electron trapping. It should also be noted that the TiN film used in this work was only 10 nm. After deposition, the surface morphology remained largely governed by the substrate topography. For this reason, only the SEM and LSCM results of the laser-textured Al_2_O_3_ substrates at laser powers of 6, 12, and 18 W after 10 nm TiN deposition are presented here (Figure 4 and Figure 5).

As shown in Figure 4a, the as-received substrate exhibits a relatively uneven machined surface without obvious laser-induced topographical features. After laser treatment at 6 W (Figure 4b), ablation-induced structures began to appear on the surface, indicating that the incident laser energy was sufficient to initiate local material removal and surface reconstruction. However, the textured morphology remained spatially non-uniform, and some areas still appeared insufficiently modified. This suggests that the laser energy density at 6 W was not high enough to produce complete and homogeneous ablation over the entire scanned region. When the laser power was increased to 12 W (Figure 4c), the surface morphology changed dramatically and was almost entirely covered by ablation-induced features. Compared with the 6 W sample, the textured layer became much more continuous and developed a rough, hierarchically reconstructed surface. Such a morphology indicates that the laser-material interaction reached a more suitable regime, in which melting, evaporation, redeposition, and repeated local restructuring collectively generated abundant surface relief. From the viewpoint of SEY regulation, this type of fully developed micro-structured surface is expected to be advantageous because it can provide more complex electron-transport pathways and a higher probability of secondary-electron recapture [15]. At a still higher laser power of 18 W, presented in Figure 4d, the surface morphology changed again. Although the surface was strongly modified, the ablation intensity became excessive, and the original coarse-textured features were largely destroyed, resulting in a comparatively finer and more homogenized surface appearance. In other words, the overly strong ablation led to excessive material removal and surface remelting, which reduced the population of distinct geometrical features beneficial for electron trapping. This observation suggests that increasing laser power does not monotonically improve the effectiveness of surface texturing. Instead, there appears to be an optimal processing window: insufficient power leads to incomplete texturing, whereas excessive power may over-remelt the surface and diminish the structural complexity required for efficient SEY suppression.

#### 3.2.2. LSCM

The three-dimensional surface topographies of the as-received and laser-textured Al_2_O_3_ substrates are shown in Figure 5. Compared with the as-received sample, all laser-treated surfaces exhibit a geometrical complexity of the substrate. For the as-received substrate, the surface is relatively flat overall, with a low arithmetic mean height Sa of 0.45 μm, indicating only limited height fluctuation. After laser treatment at 6 W (Figure 5b), the surface becomes visibly rougher, and the Sa value increases to 1.66 μm. This result suggests that the applied laser energy was sufficient to induce local ablation and surface reconstruction, but the generated features remain relatively shallow and spatially limited. When the laser power is increased to 12 W, the surface topography changes significantly and exhibits the highest degree of height variation among all samples. Correspondingly, the Sa value rises sharply to 10.01 μm, demonstrating that this condition produces the most developed and complex surface structure. When the laser power is further increased to 18 W, the surface remains rougher than the as-received substrate, but the Sa value decreases to 3.62 μm, which is much lower than that of the 12 W sample. This indicates that excessive laser power does not further enhance the surface complexity. Instead, overly strong ablation likely causes excessive material removal and local remelting, which partially destroys the previously developed coarse features and results in a comparatively finer and more homogenized surface.

Overall, both the SEM images, three-dimensional topography and roughness analysis indicate that laser ablation significantly increases the surface roughness of Al_2_O_3_ substrates, and the sample treated at 12 W exhibits the most pronounced surface reconstruction and the highest Sa value. From the viewpoint of morphology optimization for SEY suppression, this condition appears to be the most favorable among the investigated laser powers.

#### 3.2.3. XPS

**Figure 6:**
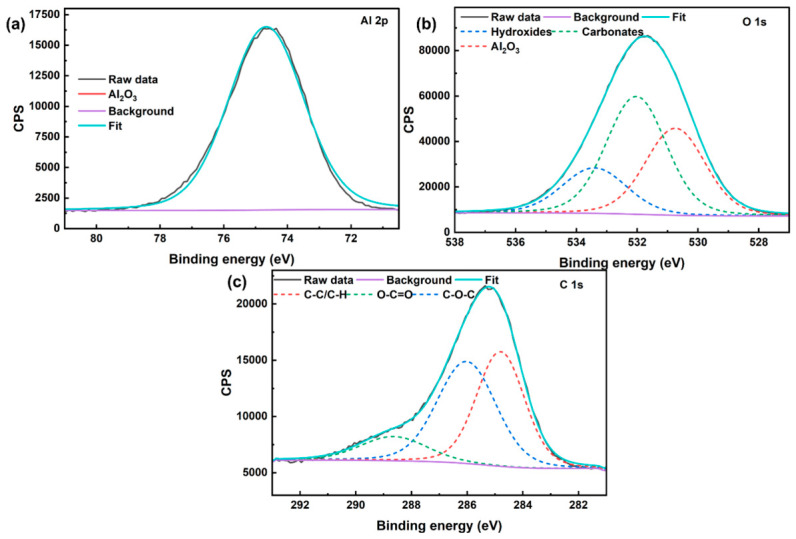
High-resolution XPS spectra of the Al_2_O_3_ substrate after laser texturing at 12 W: (**a**) Al 2p, (**b**) O 1s, and (**c**) C 1s.

To further clarify the surface chemical changes induced by laser texturing, XPS analysis was performed on the Al_2_O_3_ substrate treated at 12 W, which showed the most developed surface morphology in the SEM and LSCM results. As shown in Figure 6, the high-resolution spectra include Al 2p, O 1s, and C 1s signals, providing information on the chemical states of the laser-textured surface. In the Al 2p spectrum, only one fitted component assigned to Al_2_O_3_ is observed after laser texturing, centered at approximately 74.7 eV. Compared with the untreated sample, no metallic-Al-related contribution is detected, which may be because nanosecond laser ablation involves intense localized heating, melting, and rapid resolidification, which can strongly promote surface oxidation during or after processing. In addition, laser-induced melting and re-solidification may remove or bury the weak metallic-Al-like signal previously associated with incompletely oxidized or locally heterogeneous surface regions, leaving a more fully oxidized Al_2_O_3_-dominated surface in the XPS probing depth [27]. At the same time, the hydroxide-, carbonate-, and contamination-related species are also detected in O 1s and C 1s spectra.

**Figure 7:**
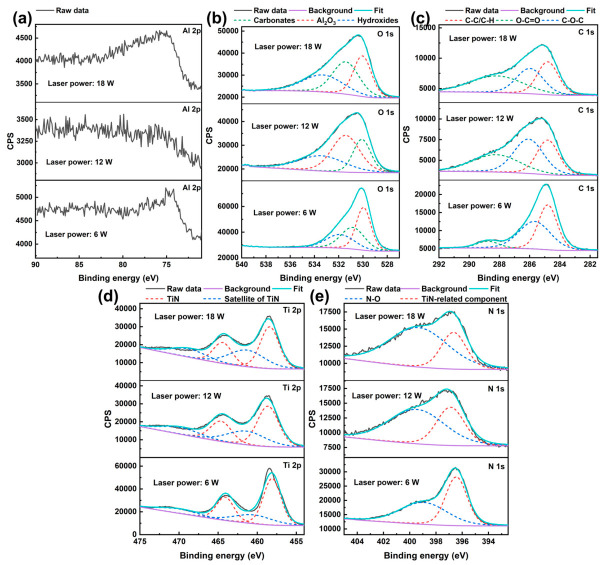
High-resolution XPS spectra of TiN films deposited at 300 W on Al_2_O_3_ substrates textured at different laser powers: (**a**) Al 2p, (**b**) O 1s, (**c**) C 1s, (**d**) Ti 2p, and (**e**) N 1s.

To further evaluate the effect of laser texturing on the subsequent deposition behavior of the TiN film, XPS measurements were performed on the Al_2_O_3_ substrates textured at 6, 12, and 18 W and then coated with TiN under the optimized sputtering power of 300 W. As shown in Figure 7, the spectra include Al 2p, O 1s, C 1s, Ti 2p, and N 1s, allowing the substrate-related and film-related chemical states to be compared for different laser-textured surfaces.

In the Al 2p spectra, no complete or pronounced Al-related peak was detected after TiN deposition for any of the laser-textured samples, indicating that the deposited TiN film effectively suppressed the substrate signal within the XPS probing depth. Notably, the sample textured at 12 W shows the strongest shielding effect, with the Al 2p signal becoming nearly undetectable. By comparison, the samples textured at 6 and 18 W still exhibit weak residual Al-related features. This result suggests that the TiN film deposited on the 12 W textured surface achieved the most effective surface coverage. Considering the morphology results discussed above, this behavior is likely associated with the more fully developed and interconnected surface texture formed at 12 W, which provides a favorable topographical basis for TiN film anchoring and coverage. In contrast, incomplete texturing at 6 W and excessive ablation/remelting at 18 W may lead to less ideal local film continuity, thereby allowing a weak substrate signal to remain detectable.

The O 1s and C 1s spectra are generally similar for all three samples: the O 1s spectra can be deconvoluted into carbonates, Al_2_O_3_ lattice oxygen, and hydroxides, while the C 1s spectra consist of C-C/C-H, O-C=O, and C-O-C components, indicating that the near-surface region still retains contributions from the oxide substrate together with adventitious carbon and oxygen-containing adsorbates introduced during air exposure. The Ti 2p and N 1s spectra clearly show TiN-related features for all three laser-textured samples, including the characteristic TiN doublet with satellite structure in Ti 2p and the TiN-related nitrogen component together with a weaker N-O contribution in N 1s, confirming that TiN films were successfully deposited on all textured surfaces and that their overall TiN chemical states are broadly comparable.

The XPS results indicate that the chemical states of the TiN-coated surfaces are broadly similar for the three laser powers, and all of them exhibit clear TiN-related features. The most notable difference lies in the degree of suppression of the substrate-related Al signal, which is strongest for the 12 W sample. This suggests that the surface textured at 12 W provides the most favorable condition for achieving effective TiN coverage. More importantly, since TiN was successfully formed on all three textured surfaces and the overall chemical-state differences among them are relatively limited, the subsequent variation in SEY is expected to be governed predominantly by the difference in surface geometry rather than by major differences in the intrinsic surface chemistry.

### 3.3. SEY Performance of Different Surfaces

**Figure 8:**
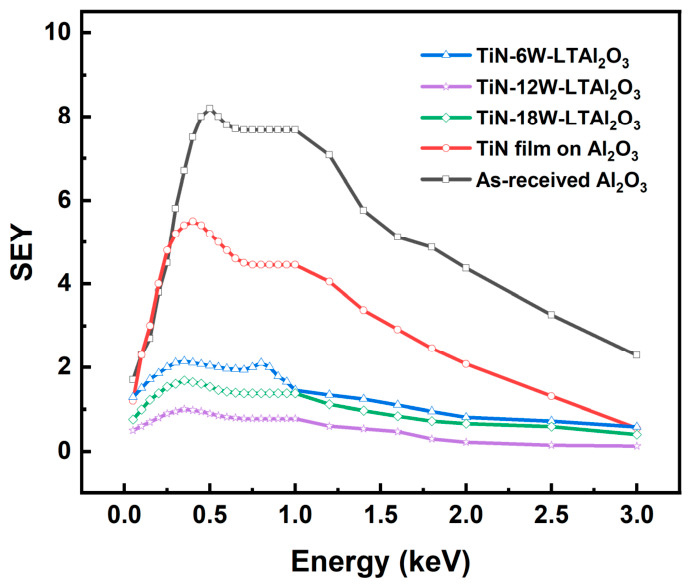
SEY curves of the as-received Al_2_O_3_ substrate, 10 nm TiN-film-coated Al_2_O_3_ substrate, and laser-textured Al_2_O_3_ substrates coated with 10 nm TiN film at different laser powers.

Figure 8 shows the SEY curves of five samples, including the as-received Al_2_O_3_ substrate, the TiN film-coated Al_2_O_3_ substrate, and the laser-textured Al_2_O_3_ substrates coated with TiN film at laser powers of 6, 12, and 18 W. The as-received Al_2_O_3_ sample exhibits the highest SEY over the whole tested energy range, with a maximum value of 8.2, indicating the intrinsically strong electron-emission tendency of the untreated ceramic surface. This result is consistent with previous studies showing that alumina-based insulating surfaces generally possess high SEY and are therefore prone to electron multiplication and multipactor-related effects in vacuum electronic and accelerator environments [7]. After depositing the 10 nm TiN film on the flat Al_2_O_3_ substrate, the maximum SEY decreases markedly from 8.2 to 5.5, demonstrating that TiN itself can effectively reduce SEY. Nevertheless, although the TiN film significantly suppresses SEY, the value remains relatively high, indicating that chemical modification alone is insufficient to achieve strong SEY suppression in the present system.

A much more pronounced reduction is observed after introducing laser surface texturing prior to TiN deposition. This result clearly demonstrates that laser texturing provides an additional and dominant suppression effect beyond that of the TiN film itself. Such behavior is well consistent with the established mechanism of structured surfaces, where geometrical features can prolong the escape path of low-energy secondary electrons, enhance multiple scattering and recapture, and thus greatly reduce the effective SEY [12]. More importantly, the variation in SEY among the three textured samples shows a clear correlation with the surface morphology revealed by SEM and LSCM. Among the three textured samples, the one processed at 12 W exhibits the lowest maximum SEY, approximately 1.0, which corresponds to the most developed surface morphology and the highest roughness (Sa = 10.01 μm), corresponding to the strongest suppression effect. This agrees well with the SEM and LSCM results, which showed that the 12 W condition produced the most developed and complex surface morphology, together with the highest roughness (Sa = 10.01 μm). Such a hierarchically reconstructed surface is expected to provide the largest number of geometrical trapping sites and the most tortuous electron-escape pathways, thereby maximizing the probability of electron re-absorption before emission into vacuum. By contrast, the 6 W sample shows a higher maximum SEY of 2.1, which can be attributed to incomplete texturing and the relatively limited development of surface structures, as observed in the SEM results. In this case, the insufficient geometrical complexity reduces the effectiveness of electron trapping. The 18 W sample, although still much better than the non-textured TiN-coated surface, shows a higher maximum SEY than the 12 W sample. This is consistent with the morphological analysis indicating that excessive laser power caused over-ablation and partial remelting, which reduced the structural hierarchy and surface roughness (Sa = 3.62 μm), thereby weakening the geometrical trapping effect. As shown in Table 3, although various low-SEY materials such as TiN and carbon-based coatings have been reported, the present work achieves a significant reduction in SEY on an Al_2_O_3_ ceramic substrate, and further demonstrates that combining TiN film deposition with laser-induced surface texturing can provide enhanced suppression performance.

### 3.4. Synergistic SEY Suppression Mechanism

**Figure 9:**
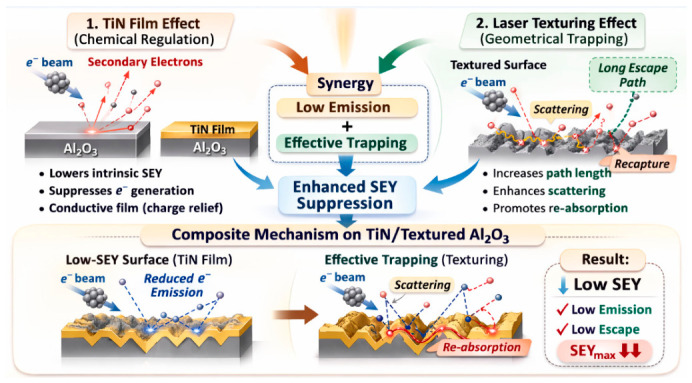
Synergistic SEY suppression mechanism by TiN film and laser surface texturing on Al_2_O_3_ ceramic.

The suppression of SEY in this work arises from the synergistic effect of the TiN film and laser surface texturing (Figure 9). These two strategies act through different but complementary mechanisms. The TiN film mainly reduces the intrinsic electron-emission ability of the Al_2_O_3_ surface, while laser texturing mainly lowers the escape probability of emitted secondary electrons by introducing geometrical trapping effects. TiN can effectively suppress secondary electron generation compared with bare Al_2_O_3_. The TiN film replaces the high-SEY ceramic surface with a material of lower intrinsic SEY. The role of laser surface texturing is to reconstruct the surface geometry and thereby alter the transport path of secondary electrons after their generation. When secondary electrons are emitted from a relatively flat surface, they are more likely to escape into a vacuum. By contrast, when the surface is covered with grooves, pores, protrusions, or hierarchically roughened structures, the emitted electrons are more likely to undergo multiple collisions with nearby sidewalls and neighboring features, leading to repeated scattering and eventual recapture. The synergistic effect originates from the fact that these two mechanisms act at different stages of the SEY process. The TiN film lowers the initial generation of secondary electrons, whereas the textured morphology suppresses the subsequent escape of those electrons, which together establish a composite surface with both lower electron-emission capability and lower electron-escape probability.

## 4. Conclusions

In this work, a composite strategy combining TiN film deposition and laser surface texturing was proposed to suppress the SEY of Al_2_O_3_ ceramic substrates. The main conclusions can be summarized as follows.

(1) TiN films were successfully deposited on Al_2_O_3_ substrates by pulsed DC magnetron sputtering, and the sputtering power had a significant influence on the film morphology and chemical states. Among the investigated conditions, 300 W provided the most favorable balance between film coverage, surface uniformity, and TiN-related chemical-state characteristics.
(2) Laser surface texturing effectively reconstructed the surface morphology of Al_2_O_3_ substrates and significantly increased the surface roughness. The sample treated at 12 W exhibited the most developed surface structure and the highest roughness, indicating the most favorable geometrical features for secondary-electron trapping.
(3) The SEY results demonstrated that both TiN film deposition and laser surface texturing contributed to SEY suppression, while their combination produced a much stronger effect. The maximum SEY decreased from 8.2 for the as-received Al_2_O_3_ substrate to 5.5 after TiN film deposition, and was further reduced to 2.1, 1.0, and 1.7 for the laser-textured TiN/Al_2_O_3_ samples prepared at 6, 12, and 18 W, respectively. Among all samples, the 12 W laser-textured TiN/Al_2_O_3_ surface exhibited the best suppression performance.
(4) The enhanced SEY suppression originates from the synergistic effect of low-SEY TiN surface chemistry and laser-induced geometrical trapping. The TiN film lowers the intrinsic electron-emission capability of the Al_2_O_3_ surface, while the textured morphology increases electron scattering, recapture, and re-absorption, thereby reducing the escape probability of secondary electrons. This synergy is the fundamental reason for the superior SEY suppression of the textured TiN/Al_2_O_3_ surface.

## Figures and Tables

**Table 1:** Summary of different samples for SEY measurements.

No.	Samples	Laser Texturing	Laser Power	TiN Film
1	As-received Al_2_O_3_	no	-	-
2	TiN-Al_2_O_3_	no	-	10 nm
3	TiN-6W-LTAl_2_O_3_	yes	6 W	10 nm
4	TiN-12W-LTAl_2_O_3_	yes	12 W	10 nm
5	TiN-18W-LTAl_2_O_3_	yes	18 W	10 nm

**Table 2:** Deposition rate of TiN films on the Al_2_O_3_ substrate at different sputtering powers.

Sputtering Power, W	100	200	300	400	500
Deposition rate, nm/min	0.55	1.85	3.85	4.4	5.9

**Table 3:** Low-SEY coating materials reported in the literature and this work.

Coating Material	Representative Substrate	SEY Performance	Refs.
TiN	Alumina ceramic	1.8	[9]
Amorphous carbon (a-C)	Accelerator vacuum chamber surface	1.05	[28]
Graphene	Stainless steel	1.4	[29]
Carbon nano-onion	Ni	0.75	[30]
Graphene	Polyimide Materials	1.52	[31]
TiN	Al_2_O_3_ ceramic	5.5	This work
TiN/textured Al_2_O_3_	Al_2_O_3_ ceramic	1.0	This work

## Data Availability

The data presented in this study are available on request from the corresponding author.
